# Digital Psychosocial Interventions Tailored for People in Opioid Use Disorder Treatment: Scoping Review

**DOI:** 10.2196/69538

**Published:** 2025-07-11

**Authors:** Madison Scialanca, Karen Alexander, Babak Tofighi

**Affiliations:** 1 Friends Research Institute, Social Research Center, 1040 Park Ave, Ste. 103, Baltimore, MD, 21201, United States, 1 4108373977

**Keywords:** mHealth, counseling, treatment, behavioral intervention, opioid treatment program

## Abstract

**Background:**

A total of 60% of patients with opioid use disorder (OUD) leave treatment early. Psychosocial interventions can enhance treatment retention by addressing behavioral and mental health needs related to early treatment discontinuation, but intervention engagement is key. If well-designed, digital platforms can increase the engagement, reach, and accessibility of psychosocial interventions. Prior reviews of OUD treatment have predominantly focused on outcomes, such as reductions in substance use, without identifying the underlying behavior change principles that drive the effectiveness of interventions.

**Objective:**

This scoping review aims to document and describe recent digital psychosocial interventions, including their behavior change strategies, for patients receiving medication for OUD (MOUD).

**Methods:**

Predefined search terms were used to search Ovid, CINAHL, and PubMed databases for peer-reviewed literature published in the last 10 years. The database search resulted in 1381 relevant studies, and 16 of them remained after applying the inclusion criteria. Studies were included if they (1) evaluated a digital intervention with behavioral, psychosocial, or counseling components for people in OUD treatment and (2) were published in English in peer-reviewed journals.

**Results:**

The 16 studies reviewed comprised 6 randomized controlled trials, 6 pilot studies, 2 qualitative studies, and 2 retrospective cohort studies. Smartphone apps (n=8) were the most prevalent intervention delivery method, with other studies using telemedicine (n=3), virtual reality (n=1), telephone calls (n=1), or text messaging (n=3) to deliver psychosocial interventions in either a synchronous (n=7) or asynchronous (n=9) manner. The digital interventions reviewed predominately delivered cognitive behavioral therapy education through a phone call (n=1), a text message (n=2), a smartphone app (n=7), or tele-counseling (n=1). The predominant behavior change strategies implemented were self-monitoring, feedback and reinforcement, psychoeducation, cue awareness, and providing instruction. One intervention reviewed uses the evidence base of mindfulness-oriented recovery enhancement.

**Conclusions:**

Participants in the studies reviewed indicated a preference for digital, flexible, patient-centered psychosocial interventions that emphasized improved patient-provider relationships. While randomized controlled trials comprised a significant portion of the studies, the inclusion of pilot studies and qualitative research highlights the field’s ongoing exploration of feasibility and effectiveness. These findings underscore the growing role of digital health solutions in psychosocial care, though further research is needed to optimize engagement, delivery, and long-term outcomes.

## Introduction

In 2020, 2.7 million people aged 12 years and older reported having an opioid use disorder (OUD) [1]. Notably, 60% of people in OUD treatment discontinue treatment early, often due to substance use cravings, negative mood, and drug cues in the environment [2]. Psychosocial support within OUD treatment can address these internal and external factors to increase treatment retention [3,4]. Several psychosocial intervention approaches exhibit a strong evidence base, including cognitive behavioral therapy (CBT) [5-7], mindfulness-oriented recovery enhancement (MORE) [8], contingency management [9], and motivational interviewing [10-12]. However, effectiveness is limited by organizational (eg, insufficient staffing, resources, and space) and patient-level barriers (eg, time, transportation, limited awareness, and stigma) [3]. Recently, personalized approaches for psychosocial support using digital methods have emerged, overcoming the multilevel constraints of in-person psychosocial services.

Substance use treatment programs have rapidly adopted digital methods to improve health care delivery, particularly during the COVID-19 pandemic and the implementation of social distancing measures [13]. Digital delivery methods, such as websites, smartphone apps, telemedicine, and text messaging, can directly address physical barriers to receiving OUD services (eg distance and lack of transportation) and extend the reach of psychosocial support services. In addition, digital health care delivery potentially addresses individual reasons for not engaging in treatment, especially among people with stigmatized conditions, such as OUD, who could benefit from increased privacy and anonymity through digital platforms [14]. However, the field of digital interventions within OUD treatment remains in a developmental phase. Therefore, we undertook a scoping review due to the absence of sufficient evidence from large randomized clinical trials, which is typically required to support the methodological rigor of a systematic review.

In addition, prior reviews of digital interventions within OUD treatment have predominantly focused on outcomes, such as reductions in substance use, without identifying the underlying behavior change principles that drive the effectiveness of interventions [15-18]. Behavior change principles refer to strategies grounded in psychological theories that help individuals modify their actions, such as reinforcement, goal setting, and self-regulation [19,20]. However, existing digital behavioral interventions for OUD have often lacked explicit integration of these principles, which may limit their effectiveness in fostering lasting behavior change [16,17]. Furthermore, previous reviews on digital interventions for OUD have overlooked the mechanisms of action and behavior change strategies used within these interventions [18,19]. For example, systematic reviews have primarily assessed treatment outcomes without fully exploring how different intervention designs incorporate or fail to incorporate behavior change principles [21,22]. This review aims to fill this gap by specifically examining how behavior change principles are used in digital interventions for OUD and by offering a more nuanced understanding of their role in improving treatment outcomes.

## Methods

### Data Sources and Search Strategy

We determined the study’s objective, the research question, inclusion and exclusion criteria, and methods a priori. The five-stage process outlined by Arksey and O’Malley [23] of the Joanna Briggs Institute was used to conduct the scoping review: (1) identify the research question, (2) identify the relevant studies, (3) select studies, (4) chart the data, and (5) organize the results [23-25]. PubMed, CINAHL, Ovid, and MEDLINE were searched with dates restricted from 2015 to 2024: (mHealth OR ecological momentary OR real-time OR mobile health technology OR digital health OR telemedicine OR text messaging OR mobile) AND (counseling OR behavioral OR psychosocial OR therapy) AND (opioid use disorder OR opioid use disorder treatment OR methadone OR buprenorphine OR naltrexone). The search term list was compiled by study team members (KA and MS). The PRISMA-ScR (Preferred Reporting Items for Systematic Reviews and Meta-Analyses extension for Scoping Reviews) guidelines (Checklist 1) were followed during the review process [26]. This scoping review did not require institutional review board approval, as it involved the synthesis of publicly available data and did not involve direct interaction with human participants or the collection of personal, identifiable information.

### Study Selection

**Figure 1.**
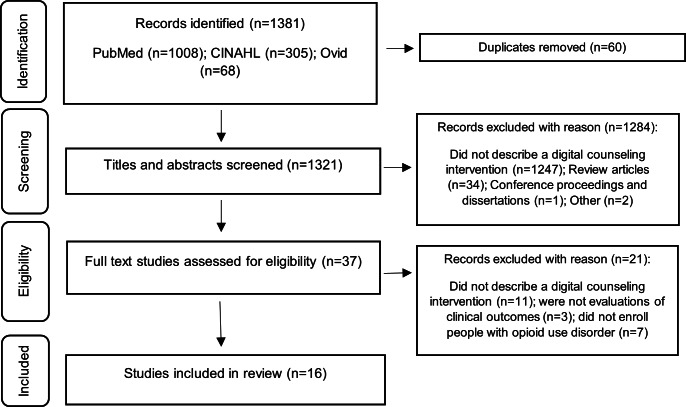
PRISMA (Preferred Reporting Items for Systematic Reviews and Meta-Analyses) flow diagram.

Studies identified in the search process were screened by title and abstract by 2 researchers (KA and MS) and selected for full review if the studies: (1) described a digital health intervention during OUD treatment, (2) also described a counseling, behavioral therapy, or psychosocial component, (3) were written in English, and (4) were original, empirical research published in peer-reviewed journals within the last 10 years. Reviews, opinion studies, and commentaries were excluded, but as this is a scoping review, no exclusion occurred due to the study design. Duplicates were removed across the databases after importing all results into a common Microsoft Excel spreadsheet. The screening process was refined prior to initiation by 2 research team members (KA and MS), and any protocol irregularities were discussed as an entire research team. The data extraction forms were piloted prior to their use to ensure clarity and consistency in the data collection process. Studies eligible for full-text review were cross-checked by 2 reviewers independently, and disagreements were discussed until consensus was achieved. To ensure accuracy and reliability, a process of data confirmation and checking was implemented, where a subset of the final dataset was independently reviewed by a third reviewer. The search process is reflected in a PRISMA (Preferred Reporting Items for Systematic Reviews and Meta-Analyses) flow diagram (see Figure 1) [27]. The search yielded 1381 studies for title and abstract review. Of the 37 studies reviewed in full text, 21 did not meet the eligibility criteria and were excluded. In all, 16 studies [16,28-42] were included in the results (See Figure 1).

### Data Extraction

Data were extracted and charted into a table format with the following headers: author, year, country, journal, study aim, study design, intervention characteristics, and outcome findings. This allowed for comparison across studies according to study characteristics. The results were then organized in the following manner: description of studies, digital health mode of delivery, principles of behavior change, the feasibility and acceptability of digital psychosocial interventions, and digital psychosocial intervention outcomes.

## Results

### Description of Studies

The 16 studies ranged in publication dates from 2016 to 2024, with most studies published in the last 2 years (n=10). Six studies [28,29,33,37,40,41] were randomized controlled trials (RCTs) with sample sizes ranging from 34 to 414. There were 6 nonrandomized pilot studies [31,32,34-36,42] that prospectively assigned participants to an intervention with sample sizes ranging from 15 to 50. The remainder of the studies were qualitative (n=2) or retrospective cohort studies (n=2). The participants in the reviewed studies were all adults aged 18 years and older. Specific inclusion criteria for the studies varied, but all studies had a common inclusion criterion of people diagnosed with OUD and treated with MOUD. Most studies were conducted in conjunction with a treatment clinic (n=15), but one study used national recruitment methods. All but 2 studies [16,30] were conducted in the United States. All reviewed studies are fully described in Table 1 below.

**Table 1.** Description of the included studies

Authors	Study purpose	Study design	Sample size and Setting (Country)	Digital health method	Type of interaction	Key findings
Ashrafioun et al [28]	To report the feasibility, acceptability, and efficacy of CBT^a^ for people experiencing loneliness and OUD^b^ compared to health education	RCT^c^	125, Community (United States)	Telehealth	Synchronous sessions with a counselor	There were significant reductions in loneliness and increases in perceived social support across both groups. The treatment group had significantly less overall opioid use and substance use from baseline to 2 mo.
Cooperman et al [29]	To evaluate the efficacy of MMT^d^ as usual (usual care) versus telehealth.MORE^e^ plus usual care among people with an OUD and pain.	RCT	154, MMT (United States)	Telehealth	Synchronous sessions with counselors	There were significant reductions in returning to drug use among MORE participants compared to treatment as usual.
Day et al [30]	To describe clinical outcomes from Alberta’s Telehealth Opioid Dependency Program	Cohort	440, OAT^f^ (Canada)	Telehealth	Synchronous counselors and providers	Participants reported high satisfaction with telehealth delivery and overall reductions in drug use and social functioning.
Garland et al [31]	To assess the feasibility of virtual reality–based MORE-VR^g^ for patients with OUD	Pilot	34, Community (United States)	Virtual Reality	Synchronous sessions with interventionists	Participants reported high usability and acceptability of MORE-VR, and opioid use decreased significantly posttreatment.
Guarino et al [32]	To pilot a mobile phone–based psychosocial intervention based in the Therapeutic Education System	Pilot	25, MMT (United States)	mHealth^h^ app	Asynchronous, not on-demand	Participants reported using the mobile intervention in a range of settings, including during times of heightened risk for substance use, and finding it helpful in managing drug cravings. Participants endorsed its usefulness and ease of use.
Gustafson et al [33]	To test a behavioral intervention to promote retention in medication treatment for OUD (A-CHESS)^i^	RCT	414, OTP^j^ (United States)	mHealth app	Asynchronous, access to on-demand content	A-CHESS did not increase opioid abstinence compared to medication treatment as usual for OUD.
Hodges et al [34]	To pilot test a mHealth app (HOPE; Warm Health Technology^k^) to improve retention and mental health outcomes	Pilot	25, OTP (United States)	mHealth app	Asynchronous, bidirectional messaging	Participants had average retention rates at the OTP and engaged consistently and positively with the mHealth app.
Kiburi et al [16]	To describe the experiences of participants with OUD enrolled in a text-messaging intervention	Qualitative	24, MMT (Kenya)	Text-messaging	Asynchronous, unidirectional messaging back to counselors	Participants described a stronger therapeutic alliance with their counselors as a result of the text message interactions.
King et al [35] (2024)	To describe the feasibility and acceptability of an mHealth app to improve treatment retention for people with OUD	Pilot	15, MMT (United States)	mHealth app	Synchronous, unidirectional messaging back to counselors	Participants rated their experience with the app (KIOS; Biomedical Development Corporation) as favorable overall. The app was able to accurately predict cravings and is designed to provide feedback to the user to have an impact on substance use.
Monico et al [36]	To evaluate the feasibility and acceptability of a digital therapeutic that combines CBT and buprenorphine telehealth treatment	Pilot	27, National recruitment (United States)	mHealth app	Synchronous telehealth visits and asynchronous content	Participants reported increased opioid abstinence days from baseline to 12 wk.
Moore et al [37]	To test the efficacy of a phone-based adjunct treatment to methadone treatment for OUD	RCT	82, OTP (United States)	Telephone	Asynchronous, on-demand content	Days of self-reported drug abstinence improved for participants in the intervention compared to treatment as usual.
Ranjit et al [38]	To examine the content of communication between participants in OUD recovery and their coaches through text-messaging intervention	Qualitative	70, OTP (United States)	mHealth app	Synchronous, bidirectional messaging	A content analysis of texts sent within the uMAT-R mHealth app. Most texts were initiated by coaches and mainly provided emotional support. Participants did engage in support-seeking behavior.
Stidham et al [39]	To examine whether treatment-related outcomes differ for young adults in treatment for OUD based on app engagement	Cohort	35, OTP (United States)	mHealth app	Asynchronous, plus contingency management rewards	There were no differences in mental health outcomes, but engagers with reSET-O (Pear Therapeutics) were more likely to be retained in care at the end of the 12 wk prescription as compared to nonengagers.
Tofighi et al [40]	To assess the feasibility of integrating text messaging into primary care–initiated buprenorphine treatment	RCT	128, OBOT^l^ patients (United States)	Text messaging	Asynchronous, unidirectional messaging	The intervention was feasible and acceptable to participants, and retention in the intervention group compared to the treatment-as-usual group did not differ significantly.
Tofighi et al [41]	To assess the feasibility of integrating text messaging into primary care–initiated buprenorphine treatment	RCT	50, OBOT patients (United States)	Text messaging	Asynchronous, unidirectional messaging	Most participants responded to at least one text query over an average of 24 d, to respond to CBT queries, confirm appointments, or ask questions about insurance.
Waselewski et al [42]	To develop and pilot test a mHealth app (HOPE) to support patients in an OTP	Pilot	25, OTP (United States)	mHealth app	Asynchronous, bidirectional messaging	Participants demonstrated engagement with the app, and qualitative analysis highlighted the value of self-monitoring to the participants.

a CBT: cognitive behavioral therapy.

b OUD: opioid use disorder.

c RCT: randomized controlled trial.

d MMT: methadone maintenance treatment.

e MORE: mindfulness-oriented recovery enhancement.

f OAT: Opioid Agonist Treatment.

g MORE-VR: mindfulness-oriented recovery enhancement-virtual reality.

h mHealth: mobile health.

i A-CHESS: Addiction-Comprehensive Health Enhancement Support System.

j OTP: opioid treatment program.

k HOPE: Heal. Overcome. Persist. Endure.

l OBOT: Office-based Buprenorphine Treatment.

### Mode of Delivery

Smartphone apps (n=8) were the most prevalent mode of delivery, with other studies using telemedicine (n=3), virtual reality (n=1), a phone (n=1), or text messaging (n=3) to deliver psychosocial interventions. Telemedicine and virtual reality interventions included synchronous participation by counselors and participants. The remaining interventions, inclusive of a telephone-based study, smartphone apps, and text-messaging interventions, used asynchronous content delivered to participants with either the ability to interact back and forth with counselors (bidirectional messaging) or a one-way method of communication only (unidirectional messaging).

### Behavior Change Techniques

The most commonly used behavior change technique (n=11) was CBT education delivered to participants through a phone call (n=1), a text message (n=2), a smartphone app (n=7), or tele-counseling (n=1). The predominant CBT strategies implemented were self-monitoring, feedback and reinforcement, psychoeducation, cue awareness, and providing instruction. A few interventions mentioned the use of coping planning and goal setting. One intervention reviewed uses the evidence base of MORE. Stress management, while not explicitly described as such, was a focus of the CBT-based interventions. The MORE intervention was explicit about using mindfulness techniques to increase coping with high-risk situations of returning to use. However, few interventions provided bidirectional feedback on performance, a cornerstone of evidence-based behavior change. The behavioral change principles underpinning each study are illustrated in Table 2.

**Table 2.** Key findings.

Authors (Year)	Key findings	Behavior change principles
Ashrafioun et al [28] (2024)	Significant reductions in loneliness and increased perceived social support across both groups; the treatment group had significantly less opioid and substance use.	CBT^a^; Social Support
Cooperman et al [29] (2024)	Significant reductions in returning to drug use among MORE^b^ participants compared to treatment as usual.	Mindfulness; Feedback and Reinforcement
Day et al [30] (2022)	High satisfaction with telehealth; reductions in drug use and improvements in social functioning.	Therapeutic Alliance; Social Support
Garland et al [31] (2024)	High usability and acceptability of MORE-VR^c^; significant reductions in opioid use posttreatment.	Mindfulness; Feedback and Reinforcement
Guarino et al [32] (2016)	Participants used the intervention during high-risk periods and found it helpful in managing cravings.	CBT; Feedback and Reinforcement; Goal Setting; Psychoeducation
Gustafson et al [33] (2024)	A-CHESS^d^ did not increase opioid abstinence compared to medication treatment as usual.	CBT; Feedback and Reinforcement; Goal Setting; Psychoeducation
Hodges et al [34] (2022)	Consistent engagement with the app; average retention rates at the OTP^e^.	CBT; Feedback and Reinforcement; Therapeutic Alliance
Kiburi et al [16] (2023)	Strengthened therapeutic alliance between participants and counselors.	Therapeutic Alliance, Social Support
King et al [35] (2024)	Participants rated the app favorably; it accurately predicted craving and aimed to impact substance use.	CBT; Feedback and Reinforcement
Monico et al [36] (2024)	Increased opioid abstinence days from baseline to 12 wk.	CBT; Feedback and Reinforcement; Psychoeducation; Therapeutic Alliance
Moore et al [37] (2019)	Increased days of self-reported drug abstinence compared to treatment as usual.	CBT; Psychoeducation
Ranjit et al [38] (2023)	Most text interactions were initiated by coaches; messages provided emotional support and facilitated support-seeking behavior.	Therapeutic Alliance
Stidham et al [39] (2024)	No differences in mental health outcomes, but app engagers were more likely to be retained in care.	Contingency Management; CBT; Feedback and Reinforcement
Tofighi et al [40] (2023)	Feasible and acceptable intervention; no significant difference in retention compared to treatment as usual.	CBT; Psychoeducation; Goal setting
Tofighi et al [41] (2022)	Most participants responded to at least one text query over an average of 24 d, including CBT responses, appointment confirmations, and insurance inquiries.	CBT; Psychoeducation; Goal setting
Waselewski et al [42] (2021)	Engagement with the app; self-monitoring was valuable to participants.	CBT; Psychoeducation; Therapeutic Alliance

a CBT: cognitive behavioral therapy.

b MORE: mindfulness-oriented recovery enhancement.

c MORE-VR: mindfulness-oriented recovery enhancement-virtual reality.

d A-CHESS: Addiction-Comprehensive Health Enhancement Support System.

e OTP: opioid treatment program.

### Feasibility, Acceptability, and Usability of Digital Counseling Interventions

A total of 11 studies [28,31,32,34-36,40-44] assessed the feasibility, acceptability, or usability of their respective intervention. Some of the measures used to determine these factors included whether the intervention was interesting and enjoyable to use, whether the material was relevant, accessible, and understandable, and how many times the intervention was accessed. When surveyed or interviewed about their preferences regarding digital interventions, participants were receptive to personalized counseling interventions to support OUD recovery via virtual reality, phone-based intervention, telemedicine, smartphone, and text-based interventions [30,31,37,41,43]. Participants in a smartphone adaptation of the Therapeutic Education System (a precursor to reSET-O [44]) reported the intervention’s feasibility in various settings, including at high-risk times of drug use to manage cravings [45]. Guarino et al [32] reported that participants found it feasible and acceptable to use a smartphone app to address drug cravings. In a formative study of the KIOS app, cravings were successfully predicted with self-reported symptoms. Participants (N=15) received specific behavioral feedback addressing patterns of symptoms and rated the app overall favorably [35].

The usefulness and ease of using a smartphone app for delivery of psychosocial interventions were reported among all age groups and educational backgrounds in the reviewed studies [32] . In qualitative interviews, participants in Project HOPE (Hope. Overcome. Persist. Endure) felt that the smartphone app improved the connection to care and communication between patients and their providers [42]. Participants were given access to the app 24 hours per day and used it on average 3 times per week. The most used features were symptom reporting and behavioral counseling messaging. In a retrospective cohort analysis of a digital therapeutic, reSET-O, participants were either highly engaged (n=10, 30% of participants completing over 90% of lessons (n=11)) or disengaged (n=24, 70% of participants completing less than 25% (n=3) of 12 lessons [39]. In a text-messaging intervention used to reinforce retention in buprenorphine treatment, most participants (n=50) responded to at least one text message that centered on the manualized medical management model (eg, self-management and CBT appointment reminders) [41].

Overall, 3 studies [16,34,38] included a community or social support interaction with a therapist or coach. Project HOPE participants (n=25) could engage bidirectionally with their counselors within the app, and the messaging feature was the most used of all the features [34]. Project HOPE implemented a community board, but it was used infrequently, likely due to the low number of participants at any one time in the app [34]. Kiburi et al [16] implemented a CBT module sent to participants as homework, with the results being sent back to a therapist. Participants expressed developing an enhanced therapeutic alliance with their therapist through the intervention. Qualitative interviews highlighted that the interaction within the intervention was important for developing the therapeutic alliance. Although not able to interact physically with the therapist (all interaction occurred over text messages), participants felt connection and support, and the text-messaged content helped them develop coping skills to handle cravings [16]. Another study used social support messages (the uMAT-R app) to develop a therapeutic relationship between participants and “e-coaches.” A qualitative content analysis of the in-app messages demonstrated that emotional support was the greatest need [38].

### Digital Psychosocial Intervention Outcomes

In total, 3 clinical trial studies [28,29,37] were reviewed, which reported decreases in opioid use compared to treatment as usual. In an RCT (N=154), MORE delivered via telemedicine significantly reduced returning to drug use and early cessation of treatment for participants enrolled in methadone maintenance treatment compared to treatment as usual [29,43]. In an RCT of a telephone-based intervention, Recovery Line, participants in the intervention group (n=40) demonstrated a significant increase in self-reported days of abstinence from drugs compared to the treatment-as-usual group (n=42) [37]. Intervention participants were given access to a phone number connected to CBT-based educational content. Text message reminders prompted participants to call the hotline, but fewer than 25% of participants accessed the Recovery Line more than 10 times and demonstrated limited engagement. However, participants who called the Recovery Line self-reported greater drug abstinence days. Significant reductions in drug use also occurred from baseline to 3-, 6-, and 12-month follow-up in a telemedicine buprenorphine clinic [30].

An RCT of a CBT intervention for perceived loneliness also demonstrated effectiveness in decreasing opioid use and substance use from baseline to 2 months [28]. However, in an RCT (n=414) of a smartphone app, A-CHESS, rates of opioid abstinence were not improved relative to opioid treatment as usual [33]. A-CHESS content and features were based on self-determination theory and addressed social support and intrinsic motivation to reduce returning to use. Finally, in a pilot study of a telehealth outpatient treatment program (Pelago-Opioid) that combined buprenorphine treatment with behavioral therapy, participants (N=27) reported increased abstinence from opioids from baseline to 12 weeks (*P*<.001) [36]. Meetings with counselors were also complementary to CBT educational modules and tracking of symptoms and progress.

Most RCTs assessed treatment retention as an outcome with findings similar to standard care. Generally, participants who demonstrated greater engagement with digital interventions were more likely to be retained in care [39]. Of note, in the HOPE study, participants lost to in-person clinic follow-up continued to engage with one or more app features [34]. In a text message–based intervention as an adjunct to buprenorphine treatment, retention in treatment did not differ between participants randomized to the intervention or treatment as usual groups (*P*=.68) [40].

## Discussion

### Principal Findings

This scoping review describes recent digital psychosocial interventions for patients receiving MOUD and yielded 16 studies [16,28-42], predominantly focused on research in the United States in the last 2 years. We collected and organized data from these studies based on the type of digital intervention used, behavior change techniques, and the evaluated outcomes. Overall, this scoping review characterized recent efforts to incorporate digital psychosocial intervention delivery methods into outpatient OUD treatment settings across several study designs.

### Study Designs

The small number of digital psychosocial interventions evaluated in RCTs is surprising, given the recent shifts in health care delivery post pandemic. However, over half of the studies reviewed were published in the last 2 years. It is likely, given the lag between conducting clinical trials and publication of the results, that there are interventions under development that are not present in the literature. It is also highly likely that findings from clinical trials conducted during the COVID-19 pandemic experienced recruitment delays, further delaying publications. For future research, equivalence trials would be the most suitable option when designing clinical trials to compare digital and in-person counseling for OUD. Instead of superiority trials, this approach would improve the feasibility and expediency of establishing efficacy for new interventions.

### Digital Intervention Types

A preference among participants for telemedicine over in-person counseling was observed, and participants agreed that the digital material was more interesting and applicable than standard counseling [37]. This finding resonates with other literature reviews, which found that patients in general medical populations and with substance use disorder are more interested in engaging with telemedicine versus in-person visits [46,47]. Telemedicine is preferred as a method that can increase the usability of psychosocial interventions as it overcomes multilevel barriers to in-person visits (eg, lack of transportation, availability and flexible scheduling of sessions, and discrimination experienced in health care settings) [48]. Numerous reviews have established patient preferences and clinical data supporting the need for “hybrid” digital strategies that extend or enhance provider counseling [15-19]. However, the interventions included in this review did not elaborate on strategies to reduce cognitive burden among patients exposed to digital platforms with multiple components. Digital tools with multiple components may often appear to be redundant or impersonal for patients and reduce engagement, thus reducing retention in treatment. Using personalized menu options, natural language processing, or graded approaches (ie, adjusting the frequency or content of prompts per specific patient inputs) may offer a more personalized strategy to engage patients across various stages of OUD treatment. Importantly, applying user experience methods ensures patient input with intervention design and delivery to improve usability and longitudinal engagement [49]. The Technology Acceptance Model also reinforces a theoretically driven approach to applying patient feedback to inform intervention design and iterative refinement (eg, perceived usefulness, ease of use, relevance, and intrusiveness) that may further enhance adoption [50].

Only two of the digital interventions (KIOS and A-CHESS) reviewed used real-time data to impact substance use or treatment retention outcomes [33,35,51]. Treatment incorporating real-time data has provided an avenue for tailored counseling sessions [44]. Visualization of data on a web- or mobile-based dashboard enables counselors to focus sessions on specific triggers of drug use [52]. Counseling sessions that incorporate real-time data to respond to the unique symptoms and triggers of a particular behavior can personalize treatment [20,53,54]. For instance, leveraging ecological momentary assessments and passively captured biofeedback data through a growing array of sensors provides innovative strategies to evaluate complex, temporally dynamic psychological and physiological factors that influence treatment outcomes. However, few of the interventions reviewed were able to offer real-time support involving patient-provider communications apart from asynchronous, bidirectional messaging with counselors. Patients rated messaging favorably and engaged most often with the digital tools that enhanced their patient-provider relationship, but real-time response was rare. Real-time support is an important aspect of digital tools as it complements current models of behavior change.

### Behavior Change Techniques

The digital interventions reviewed predominately delivered CBT education and increased contact with providers. The evidence base of CBT as a behavior change technique is clear in the literature, as the primary targets of CBT (stress, coping, and problem solving) are key mechanisms involved in returning to use. CBT aims to help patients identify situations that may present an opportunity to return to use and learn to cope with these situations. However, longitudinal engagement with CBT-based digital interventions remains a persistent challenge. Here, the merging of evidence-based behavior change strategies offers a tailored approach to improve engagement with digital interventions. For instance, the MORE approach merges efficacious elements of mindfulness, cognitive behavioral therapy, and positive psychology to improve treatment retention by targeting conditions (eg, cravings, physical pain, and emotional distress) that drive illicit opioid reuse and early cessation of buprenorphine. Patient engagement with behavior change interventions early in treatment may also be enhanced using contingency management or motivational enhancement therapy-driven tools.

### Outcomes

The digital psychosocial interventions reviewed appeared feasible and acceptable for patients receiving MOUD. Interestingly, participants in one study continued the use of the HOPE app even after in-person disengagement with treatment, indicating that digital approaches may be a needed adjunct to treatment when barriers exist to in-person clinic attendance [42]. These findings suggest the need for more personalized approaches to support patients across the OUD care cascade in the event of illicit opioid reuse and preferences for receiving harm reduction content or opting for medication-free approaches to treating OUD. Three RCTs reported decreased opioid use compared to treatment-as-usual groups among patients prescribed MOUD, and one pilot study reported decreased opioid craving while using a smartphone app based on cognitive behavioral therapy. A-CHESS, which was based on self-determination theory, did not result in increased opioid abstinence. This suggests that further research is needed into the specific content or design features that effectively reduce substance use.

While current digital health interventions in the treatment of OUD show promising findings in improving engagement in care, there are significant gaps in the evidence regarding how to best adapt and integrate these digital tools into existing OUD treatment settings. Many interventions have been designed and tested in isolation, often without sufficient consideration for the complexities of real-world clinical environments. As a result, it remains unclear which specific digital tools or approaches are most effective when scaled and adapted to diverse clinical workflows. In addition, the integration of digital tools into treatment practices can be challenging for health care providers, particularly in terms of workflow disruption, time management, and the increasing burden of managing new technology alongside traditional patient care. Without further research into the nuanced needs of clinicians, the widespread adoption of these tools could face resistance or fail to achieve their full potential in improving patient outcomes.

### Limitations

Several limitations may have affected the review findings. Across the 16 studies [16,28-42], intervention mode of delivery, control groups, and treatment frequency and duration all varied, making it difficult to synthesize the findings. In addition, all but 2 studies [16,30] were conducted in the United States, one in Canada, and one in Kenya, suggesting that further research is needed in low- and middle-income countries. Similarly, these studies were only peer-reviewed published studies in English, which continues to limit the results. Although not an exclusionary criterion, it is valuable to note that there were no studies with participants who were children younger than 18 years. We also did not include any studies that used wearables or social media interventions since they were not integrated into MOUD treatment and did not implement evidence-driven behavior change models. However, future studies are needed to evaluate these modalities and other digital tools that may leverage passive data to better guide digital intervention design.

### Conclusion

Digital psychosocial interventions, particularly those delivered via smartphone apps, are increasingly used and predominantly focus on CBT education and enhancing provider contact. The findings in our scoping review underscore the growing role of digital health solutions in addiction care, though further research is needed to optimize engagement, delivery, and long-term outcomes. Future research should focus on optimizing engagement strategies to ensure sustained user participation. Digital health innovations in OUD treatment often exacerbate the digital divide, particularly for underserved patient populations who may not have access to the necessary devices or high-speed internet required for effective use of these interventions. This gap highlights the need for more inclusive and accessible digital solutions, ensuring that the benefits of technological advancements are available to all patients, regardless of socioeconomic status. Future research initiatives should focus on developing and testing more accessible digital health solutions, including low-cost devices and internet-free options, that can reach those who are most in need of care.

## Supplementary material

10.2196/69538 Checklist 1 PRISMA-ScR (Preferred Reporting Items for Systematic Reviews and Meta-Analyses extension for Scoping Reviews) checklist.

## Abbreviations

CBT: cognitive behavioral therapy
HOPE: Hope. Overcome. Persist. Endure
MORE: mindfulness-oriented recovery enhancement
MOUD: medication for opioid use disorder
OUD: opioid use disorder
PRISMA: Preferred Reporting Items for Systematic Reviews and Meta-Analyses
PRISMA-ScR: Preferred Reporting Items for Systematic Reviews and Meta-Analyses extension for Scoping Reviews
RCT: randomized controlled trial
